# Low-Dose Versus Standard-Dose Radioiodine Therapy in Differentiated Thyroid Cancer: Focus on Tolerability in a Retrospective Evaluation

**DOI:** 10.3390/ph18040443

**Published:** 2025-03-21

**Authors:** Laura Musso, Cristina Maltese, Giulio Beretta, Ilaria Patelli, Stefano Raffa, Arnoldo Piccardo, Francesco Fiz, Lara Vera, Manuela Albertelli, Michele Minuto, Diego Ferone, Marcello Bagnasco, Stefano Gay

**Affiliations:** 1 Endocrinology Unit, Department of Internal Medicine and Medical Specialties (DIMI), University of Genoa, 16132 Genoa, Italy or laura_mge@tiscali.it (L.M.); ilariapatelli94@gmail.com (I.P.); manuela.albertelli@unige.it (M.A.); diego.ferone@unige.it (D.F.); bagnasco@unige.it (M.B.); 2 Clinica Oculistica, Department of Neurosciences, Rehabilitation, Ophthalmology, Genetics, Maternal and Child Health (DiNOGMI), University of Genoa, 16132 Genoa, Italy; cris.maltese96@gmail.com; 3 Division of Pediatrics, “F. Del Ponte” Hospital, University of Insubria, 21100 Varese, Italy; giulioberetta95@gmail.com; 4 Nuclear Medicine, IRCCS Ospedale Policlinico San Martino, 16132 Genoa, Italy; stefanoraffa@live.com; 5 Department of Health Sciences (DISSAL), University of Genoa, 16132 Genoa, Italy; 6 Department of Nuclear Medicine, Ospedali Galliera, 16128 Genoa, Italy; arnoldo.piccardo@galliera.it (A.P.); francesco.fiz@galliera.it (F.F.); 7 Endocrinology Unit, IRCCS Ospedale Policlinico San Martino, 16132 Genoa, Italy; lara.vera@hsanmartino.it; 8 Endocrine Surgery Unit, IRCCS Ospedale Policlinico san Martino, 16132 Genoa, Italy; michele.minuto@unige.it; 9 Department of Surgical Sciences (DISC), University of Genoa, 16132 Genoa, Italy

**Keywords:** differentiated thyroid cancer, radioiodine therapy, clinical adverse events

## Abstract

**Background:** The role of radioiodine (RAI) therapy for differentiated thyroid cancers (DTCs) is still a matter of debate. Low-dose RAI (LDRAI) therapy is a possible treatment for patients at low–intermediate risk of recurrence. The aim of this study was to evaluate the occurrence of post-RAI therapy clinical and biochemical side effects with respect to its dosage. **Methods:** We retrospectively examined 142 patients who had been administered RAI therapy for DTCs and carried out at least a 12-month follow-up. The incidence of clinical adverse events (CAEs: xerophthalmia, xerostomia, and dysgeusia) and values for hemoglobin (Hb), red blood cells (RBCs), white blood cells (WBCs) and platelets (PLTs) during the first year of follow-up were compared between patients who underwent standard-dose RAI (SDRAI) therapy and LDRAI therapy. **Results:** Of the 142 patients, 66 were treated with LDRAI and 76 with SDRAI. A higher incidence of CAEs was found in the SDRAI group than in the LDRAI group (*p* = 0.002). An administered dose above 2849 MBq was associated with CAEs (sensitivity 88.89%, specificity 54.03%, *p* < 0.001). We found a slight decrease in Hb (*p* = 0.008), RBCs (*p* = 0.013), WBCs (*p* = 0.004) and PLTs (*p* < 0.001) in the SDRAI group, while in the LDRAI group only WBCs showed a minimal decrease (*p* = 0.027) with any occurrence of overt bone-marrow disease. **Conclusions:** According to our data, LDRAI therapy seemed to be associated with a lower incidence of CAEs than SDRAI therapy. Both methods showed an excellent safety profile in terms of hematopoietic effects. However, the effect of SDRAI therapy in this setting might have been more positive than that of LDRAI therapy.

## 1. Introduction

Differentiated thyroid cancers (DTCs) are the most common endocrine neoplasms, representing 3–4% of all tumors [1,2], and they are generally associated with very low disease-specific mortality and excellent overall survival [3]. To date, surgery represents the first line treatment of DTCs, followed by radioiodine (131-I) (RAI) administration according to the patient’s risk profile [4,5]. In the past 15 years, with the advancement in detection technology and treatment operations, thyroid cancer can be detected and treated at an earlier stage, and surgery is often sufficient to achieve remission. Nevertheless, in selected cases, RAI treatment remains advisable. Tumor-staging and measuring the tumor marker thyroglobulin allow for risk-adapted stratification [6]. Assessment of post-operative disease status is required to optimize proper patient selection for RAI therapy for remnant ablation, adjuvant treatment, or treatment of known disease [7,8]. If RAI remnant ablation is performed after total thyroidectomy for low-risk thyroid cancer, a lower dose of radioiodine (1110–1850 MBq, namely 30–50 mCi) can be administered. When RAI therapy is intended for initial adjuvant therapy, conversely, higher doses, generally 2960 to 3700 MBq, up to 5550 MBq (80 to 100 mCi, up to 150 mCi) are recommended [4,9,10].

RAI therapy is usually well tolerated and only in few cases are side effects reported. In these cases, patients experienced more frequent salivary and lacrimal gland dysfunction or transient male or female gonadal dysfunction. Pulmonary and bone-marrow affections are extremely rare and limited to patients receiving multiple RAI administrations [11,12,13,14,15].

This study’s aim was to compare the incidence of xerostomia, xerophthalmia, and dysgeusia during the first year after RAI therapy in patients treated with low-dose RAI (LDRAI) versus standard-dose RAI (SDRAI) regimens. A further endpoint was to assess the degree of bone-marrow impairment according to the regimen adopted.

## 2. Results

Of the 142 patients enrolled in the study a prevalence of females was described (112, 78.8%), and the mean age was 51.7 years (±14.4). No difference was reported between the two study groups (SDRAI and LDRAI) regarding age (52 vs. 51 years, *p* = 0.577).

In particular, 93 patients (65.5%) were treated with total thyroidectomy. At the same time, a lymph node dissection of the central compartment of the neck was associated with total thyroidectomy in 38 cases (26.8%), and a central and lateral neck compartment dissection associated with total thyroidectomy was performed in 11 patients (7.7%).

Histology revealed a papillary thyroid cancer (PTC) in 131 patients (92.3%). Of these, 17 had a follicular variant papillary thyroid cancer (FvPTC), and 4 (2.8%) patients displayed both a PTC and a follicular thyroid cancer (FTC). An FTC alone was diagnosed in 7 patients (4.9%).

Post-surgical staging yielded the following results: 97 patients (68.3%) displayed stage I, 40 (28.2%) stage II, and 5 (3.5%) stage III disease. Patients’ characteristics are summarized in Table 1.

Median post-surgery basal Tg was 0.50 ng/mL (range 0.04–48.00) in the SDRAI group and 0.04 ng/mL (range 0.04–4.53) in the LDRAI group (*p* < 0.001). Median post-surgery stimulated Tg was 2.90 ng/mL (range 0.04–140.00) in the SDRAI group and 0.11 ng/mL (range 0.04–19.00) in the LDRAI group (*p* < 0.001).

Administered RAI dose was related to the TNM stage (*p* = 0.029) and post-surgery Tg (*p* = 0.01), but not to the histology (*p* = 0.851).

**Figure 1:**
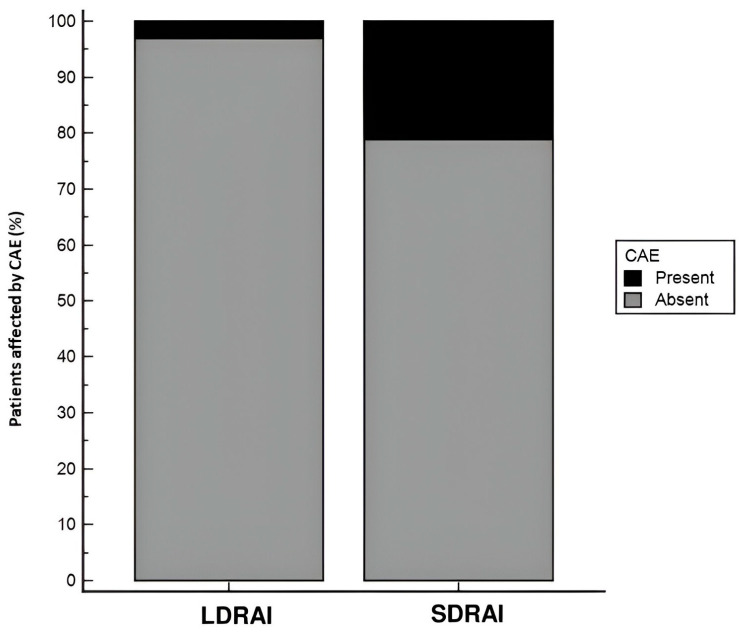
Proportion of CAEs in the two study groups (LDRAI and SDRAI). A significant prevalence of CAEs was documented in the SDRAI group (*p* = 0.002).

Overall, CAEs occurred in 18 individuals (12.7%), of whom 16 had undergone SDRAI while 2 had undergone LDRAI. In addition,13 cases of dysgeusia, 3 of xerophthalmia, and 4 of xerostomia were reported. A prevalence of CAEs was reported in the subgroup treated by SDRAI (*p* = 0.002, Figure 1). In all cases CAEs were transient and spontaneously reversible.

In the SDRAI group, 4 (5.26%) patients developed xerostomia, 2 (2.63%) xerophthalmia, and 12 (15.78%) dysgeusia.

In the LDRAI group, one (1.51%) patient developed xerophthalmia, one (1.51%) developed dysgeusia, while none developed xerostomia.

The median duration of CAEs was 3.5 months (range 2–60) for xerostomia, 4 months (range 3–12) for xerophthalmia, and 5 months (range 2–24) for dysgeusia.

No statistically significant correlation was found between each CAE and any of the parameters considered. Considering the total number of CAEs, a significant relationship was found with tumor stage; in particular, CAEs occurred in 8 of 95 patients with stage I disease (12%), 7 of 40 patients with stage II disease (18%), and 3 of 5 patients with stage III disease (60%), *p* = 0.002, while no correlation was demonstrated with patient age (*p* = 0.130), gender (*p* = 0.667), histology (*p* = 0.504), or the type of surgery performed (*p* = 0.598).

**Figure 2:**
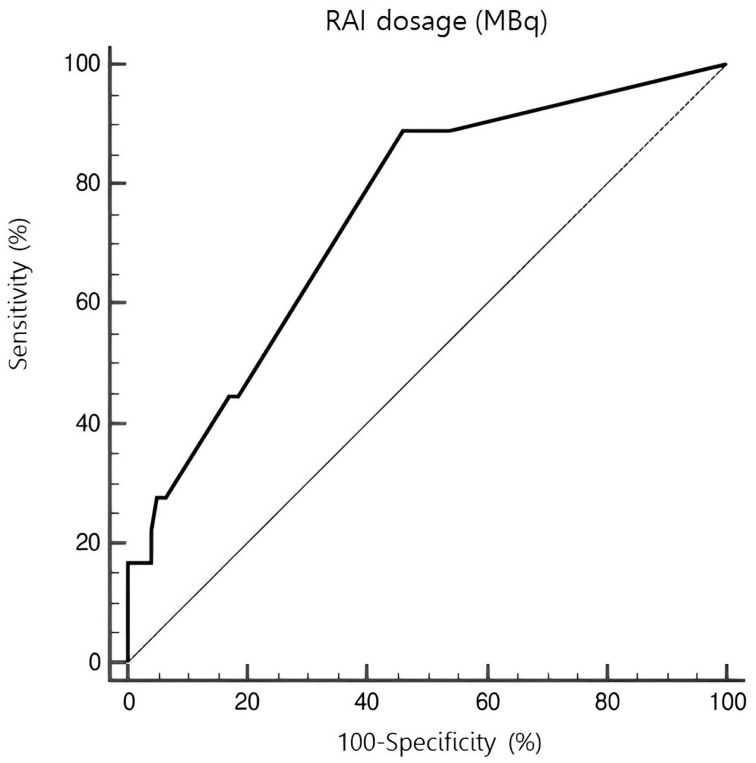
ROC curve representing the amount of radioiodine activity associated with the occurrence of CAEs. An administered dose exceeding 2849 MBq was associated with CAEs with a sensitivity of 88.89% and specificity of 54.03% (*p* < 0.001).

Using a ROC curve (Figure 2), the threshold dose of radioiodine that was most predictive of the occurrence of CAEs in our case series was evaluated. According to this, the onset of CAEs was associated with RAI dosage above 2849 MBq with a sensitivity of 88.89% and specificity of 54.03% (*p* < 0.001).

The values for Hb, RBCs, WBC, and PLTs before RAI therapy and 1 year after it in the two study groups are reported in Table 2.

As regards the pre-treatment evaluation, the LDRAI group seemed to display slightly lower RBC and WBC values.

Conversely, at the post-treatment evaluation, no significant difference was demonstrated between the two groups.

Considering the longitudinal variation in each parameter in the same group, we reported a significant decrease in Hb (*p* = 0.008), RBCs (*p* = 0.013), WBCs (*p* = 0.004), and PLTs (*p* < 0.001) in the SDRAI group, while in the LDRAI group, only WBCs showed a minimal decrease (*p* = 0.027) and no significant difference was found for Hb (*p* = 0.747), RBCs (*p* = 0.702), or PLTs (*p* = 0.576) between the first and the second evaluation.

No correlation was shown between the modifications in blood count values and age at diagnosis (*p* = 0.953 for Hb, *p* = 0.664 for RBCs, *p* = 0.869 for WBCs, *p* = 0.149 for PLTs), gender (*p* = 0.537 for Hb, *p* = 0.531 for RBCs, *p* = 0.497 for WBCs, *p* = 0.595 for PLTs), type of surgery (*p* = 0.346 for Hb, *p* = 0.966 for RBCs, *p* = 0.994 for WBCs, *p* = 0.900 for PLTs), histology (*p* = 0.888 for Hb, *p* = 0.892 for RBCs, *p* = 0.965 for WBCs, *p* = 0.240 for PLTs), or stage of disease (*p* = 0.273 for Hb, *p* = 0.226 for RBCs, *p* = 0.264 for WBCs, *p* = 0.178 for PLTs).

A significant reduction in both basal and rhTSH-stimulated Tg was observed in both groups of patients treated with the two different RAI regimens. Specifically, patients treated with SDRAI had a change in median basal Tg (0.1 vs. 0.5 ng/mL, *p* < 0.001) and stimulated Tg (0.13 vs. 2.65 ng/mL, *p* < 0.001); also, patients treated with LDRAI had a change in median basal Tg (0.04, range 0.04–3.20 ng/mL vs. 0.04, range 0.04–0.54 ng/mL; *p* = 0.002) and stimulated Tg (0.04 vs. 0.06 ng/mL, *p* = 0.001). On the other hand, Tg reduction proved more consistent in those who received SDRAI (*p* = 0.004 and *p* < 0.001 for basal and stimulated Tg, respectively).

A thyroid cancer recurrence was found in 10 patients, 9 of whom had multiple administrations of RAI (median cumulative dose 300 mCi, range 180–730 mCi). All relapsing patients had received SDRAI as their first treatment.

## 3. Discussion

The objective of the study was to evaluate the incidence of CAE among patients undergoing SDRAI and LDRAI therapies.

There was a statistically significant difference between SDRAI and LDRAI therapies, with a higher incidence of CAEs in the SDRAI group. No significant correlation was shown between the occurrence of CAEs and age, sex, tumor histology, or type of surgery. A significant correlation was, however, observed with the stage of disease; this was related to the fact that patients with more advanced disease received higher dosages of RAI.

Regarding the hematologic impact of RAI, we must clarify that in none of the treated patients did it have a clinically significant impact on bone-marrow function. On the other hand, a slight but significant effect was noted for all the variables considered (Hb, RBCs, WBCs, and PLTs) in patients undergoing SDRAI therapy, while only WBCs showed a minimal decrease in those undergoing LDRAI therapy. No correlation was shown between the change in hematological profile and age at diagnosis, sex, type of surgery, histology, or stage of the disease.

Several studies conducted on patients undergoing RAI treatment have found results similar to those reported in this paper.

With regard to the effects of RAI treatment on exocrine glands, in 2017 Moreddu and colleagues evaluated the occurrence of these effects in a cohort of 800 patients (RAI dose = 3700 MBq) through the use of a specifically designated questionnaire. Of these patients, 12% reported the occurrence of dysgeusia and 36% reported the occurrence of xerostomia. About half of the patients developed the side effects within the year after treatment (11% in the first week, 10% in the first month, 12% within the first 6 months, and 12% after the first 6 months) [16].

Hyer and colleagues reported similar complication rates in 2007 (median RAI dose = 13,986 MBq), with an overall incidence of CAEs of 26% and about a third of patients having symptoms continue for over a year after therapy [17].

A recent study [18] on 136 DTC patients showed no difference six months after RAI therapy in the level of parotid gland pain or the number of patients with hyposalivation. Still, there were significantly more patients with dry-mouth sensation and dry eyes after therapy than baseline.

More generally, studies estimate the risk of salivary side effects to be around 20–40% [19,20,21]. In our analysis, the CAE rate proved lower. We have to underline that the studies we have reported were all conducted quite a long time ago. In recent years, growing attention to this aspect and specific pre-treatment measures may have led to the anticipation and prevention of many of these effects.

The literature is less rich regarding the occurrence of xerophthalmia following RAI therapy. In 2001, Solans and colleagues analyzed a cohort of 79 patients (RAI dose range 925–18,500 MBq); tear function was studied using the Shimmer test and the Rose Bengal test, while salivary function was studied through the use of technetium (Tc-99m pertechnetate) scintigraphy. This study showed that the occurrence of subjective xerophthalmia had an incidence of 25.3%, while an objectifiable form occurred in 17.7% of patients at one year after treatment; the occurrence of subjective xerostomia, on the other hand, had an incidence of 32.9%, while it was objectifiable in 50.6% of patients at one year after treatment [22].

Da Fonseca and colleagues reported an incidence of lacrimal-system obstruction of 6.88% (3 cases in 44 patients) after RAI for DTC [23].

More recently, a large study involving 807 patients (mean age 47.8 ± 16.9 years), half of whom underwent single-dose RAI therapy (mean dose 4033 ± 1591 MBq), was carried out: 10.5% of them developed nasal symptoms and 9.7% ocular symptoms within 11 days and 10 months, respectively [24].

Lastly, a literature review reported a frequency of nasolacrimal duct obstruction ranging from 2.2% to 18% following RAI. It was mostly bilateral and noted in patients who had received an RAI dose of more than 5550 MBq [25].

Regarding the hematologic impact of RAI therapy, Prinsen and colleagues evaluated the bone-marrow response in patients undergoing RAI therapy for DTCs in 2015. The 331 patients recruited, after the average administration of 5550 MBq of RAI, developed a transient reduction in the hematologic values considered, but none of them required medical intervention due to these alterations [26].

Molinaro and colleagues, on the other hand, evaluated the occurrence and duration of cytopenia in a cohort of 206 patients after a single administration of RAI (mean dose 4662 MBq), demonstrating how it correlated with therapy and persisted for at least one year after treatment [27].

Other studies have demonstrated a small but statistically significant reduction in hematologic parameters after RAI therapy, although in these cases the dose administered was higher (mean dose > 9250 MBq) [28,29,30].

Duskin-Bitan and colleagues also obtained similar results in a study involving 122 elderly patients (age > 70 years, mean 76 ± 4.3 years) who received an average dose of 6793.2 ± 4884 MBq; none of the patients developed clinically relevant cytopenia [31].

As regards our investigation, we have to clarify that data were collected retrospectively during a quite large time period. The selection of RAI regimen was affected by different variables and particularly by the year of treatment and the current guidelines of that period. On the other hand, the main objective of the study was not to compare the effectiveness of SDRAI and LDRAI therapies in terms of disease recurrence nor to investigate the appropriacy of each regimen with respect to disease status, but to evaluate the relationship between the amount of RAI and CAEs

We also acknowledge some limitations of our study, mostly represented by the numerosity of the study population and the retrospective design of the investigation. Moreover, CAEs were evaluated as reported by patients at the time of clinical examination without using any specific test and using a self-reported binary evaluation rather than a validated scale of severity.

From our results, we may conclude that based on the higher incidence of adverse events in patients treated with SDRAI compared with those treated with LDRAI, different RAI regimens might have a different impact on a patient’s quality of life. At the same time, the different RAI regimens appears to have had a minimal effect on bone-marrow activity. On the other hand, few differences in efficacy were highlighted between SDRAI and LDRAI therapies if patients underwent a proper stratification of the risk of recurrence.

Because of this, it seems justified to stratify patients as well as possible and to select carefully the most appropriate RAI regimen to avoid the adverse effects of treatment and avoidable costs. It should certainly be remembered that if the indication exists, the beneficial effects of RAI therapy are significantly greater than the associated risks, which, therefore, should not preclude it.

## 4. Materials and Methods

The study was designed as retrospective and observational. A total of 142 patients were recruited among those affected by DTCs in follow-up at the IRCCS AOU Policlinico San Martino in Genoa and who received primary treatments between 2000 and 2020. All these patients had a follow-up of not less than 12 months to evaluate clinical adverse events (CAEs) incurred during that period.

Inclusion criteria were as follows: patients ≥ 18 years of age with a diagnosis of DTC who had undergone total thyroidectomy followed by RAI therapy, who had a follow-up duration of at least 12 months from the end of primary treatments, and who had given informed consent for data collection and processing for research purposes.

All subjects were followed by a multidisciplinary team of endocrinologists and nuclear physicians through a series of visits at least every six months, including periodic blood and ultrasound checks.

We collected medical, clinical, biochemical, and histological data through the review of medical records, and we analyzed them anonymously using unified data-collection programs. The variables analyzed in the study were personal data; type of surgery; histological reports; post-surgical staging (according to 8th edition of TNM AJCC); RAI dose and further cycles of RAI; the occurrence and duration of xerostomia, dysgeusia, and xerophthalmia during the first year of follow-up; and the values for hemoglobin (Hb), red blood cells (RBCs), platelets (PLTs), and white blood cells (WBCs) pretreatment and a year after it.

CAEs were reported as dichotomic variables (present or absent) during the follow-up. They were analyzed singularly and as a cumulative variable, namely the occurrence of at least one of the three.

Basal and stimulated thyroglobulin (Tg) pre-RAI therapy and antithyroglobulin antibody (TgAb) values were also collected.

Patients were classified into two groups based on the type of RAI regimen received: SDRAI (76 patients) or LDRAI (66 patients, 89% of them treated with 1110 MBq). Doses of RAI less than or equal to 1850 MBq were considered LDRAI. TSH stimulation was obtained by LT4 withdrawal in 49 patients (3 LDRAI and 46 SDRAI) or by rhTSH stimulation in 89 patients (62 LDRAI and 27 SDRAI). The modality of TSH stimulation was not reported in four cases.

The relationship between RAI regimen administered and the occurrence of CAEs during the first year of follow-up and their duration was analyzed.

We evaluated, in the two study groups (SDRAI and LDRAI), the trends of Hb, RBCs, PLTs, and WBCs before and 12 months after RAI therapy. Then, the relationship between RAI regimen administered and changes in the hematological profile of patients was analyzed. The occurrence of CAEs and the effect on hematological parameters after one year were then correlated with the patient-related surgical and histological variables.

As regards laboratory evaluations, serum Tg was assayed through immuno-chemiluminescence (Roche Diagnostics, Mannheim, Germany). Functional sensitivity of the method was ≤0.04 ng/mL. TgAb values were determined through fluorometric enzyme-linked immunoassay (Feia).

Regarding statistical methods, the parametric distribution of the data was assessed using the Kolmogorov–Smirnov test. Data are reported in the text as “median, range” if non-parametric, as “mean ± standard deviation” when parametric. The Fisher’s exact test was used to evaluate the association between the occurrence of CAEs and RAI regimen. The threshold dose of RAI and cut-off values for the best sensitivity and specificity in predicting the occurrence of CAEs were calculated using ROC curves. To compare the trend of the hematological parameters between LDRAI and SDRAI therapies, the longitudinal variation in each parameter was assessed using the Wilcoxon test for non-parametric data; paired samples T-test was used instead for parametric data. Statistical analyses were conducted using MedCalc Portable Launcher software, version 2.2.0.0; the same program was used to create all figures and graphs.

## 5. Conclusions

In conclusion, our work supports the evidence that, in low-risk patients, the choice of RAI regimen should also be focused on avoiding overly aggressive therapies. This may lead to a decreased incidence of adverse events.

The results of this study suggest that, after adequate stratification of patients, LDRAI therapy appears to be associated with a lower incidence of adverse events in terms of xerostomia, xerophthalmia, and dysgeusia.

Moreover, even if none of the RAI regimens can be considered potentially harmful for the bone marrow, this aspect should not be totally neglected at the time of the choice of treatment.

## Figures and Tables

**Table 1:** Characteristics of patients in the study population.

Characteristics	No. of Patients
**Gender**	
Male	30 (21.2%)
Female	112 (78.8%)
**Age at diagnosis**	
Mean	51.7 years (±14.4)
**Histology**	
PTC (classic/follicular variant)	131 (92.3%)
Follicular thyroid carcinoma	7 (4.9%)
PTC + FTC	4 (2.8%)
**Stage**	
I	97 (68.3%)
II	40 (28.2%)
III	5 (3.5%)
**Extent of surgery**	
Total thyroidectomy	93 (65.5%)
Total thyroidectomy + central compartment neck dissection	38 (26.8%)
Total thyroidectomy + central and lateral compartment neck dissection	11 (7.7%)
**RAI dose**	
LDRAI	66 (46.5%)
SDRAI	76 (53.5%)

**Table 2:** Hematological parameters (Hb, RBCs, WBCs, and PLTs) before RAI therapy and 1 year after it in the two study groups (LDRAI and SDRAI).

	LDRAI		SDRAI	
	Before RAI	1 Year After RAI	Before RAI	1 Year After RAI
Hb (g/dL)	13.61 (±1.37)	13.42 (±1.11)	13.94 (±1.66)	13.54 (±1.27)
RBCs (×10^6^/μL)	4.6 (3.9–6.1)	4.6 (3.7–5.6)	4.8 (3.0–5.8)	4.7 (3.7–8.4)
WBCs (×10^6^/μL)	6.1 (3.2–10.9)	6.3 (4.3–14.0)	7.0 (3.4–11.0)	5.8 (2.3–12.0)
PLTs (×10^3^/μL)	250 (120–441)	243 (144–495)	255 (139–453)	238 (127–425)

## Data Availability

The datasets employed or analyzed in the present study are accessible upon reasonable request from the corresponding author.

## Abbreviations

The following abbreviations are used in this manuscript:

DTC	Differentiated thyroid cancers
LDRAI	Low-dose RAI
SDRAI	Standard-dose RAI
CAE	Clinical adverse event
Hb	Hemoglobin
RBC	Red blood cell
WBC	White blood cell
PLT	Platelet
Tg	Thyroglobulin
TgAb	Antithyroglobulin antibody
